# Implementation of high‐throughput non‐invasive prenatal testing for fetal *RHD* genotype testing in England: Results of a cross‐sectional survey of maternity units and expert interviews

**DOI:** 10.1111/tme.12702

**Published:** 2020-06-11

**Authors:** Edyta Ryczek, Judith White, Grace Carolan‐Rees

**Affiliations:** ^1^ Cedar, Cardiff and Vale University Health Board, Cardiff Medicentre Heath Park Cardiff UK

**Keywords:** antenatal anti‐RhD prophylaxis, anti‐D, cffDNA, fetal RhD determination, non‐invasive prenatal testing, RAADP

## Abstract

**Background:**

Previously, routine antenatal anti‐D prophylaxis (RAADP) was administered to all RhD‐negative mothers to reduce the risk of sensitisation in the UK's National Health Service (NHS). If the baby is RhD‐negative, RAADP is not required. In 2016, the UK National Institute for Health and Care Excellence (NICE) recommended non‐invasive prenatal testing (NIPT) for fetal *RHD* genotype as a cost‐effective option to guide RAADP.

**Objectives:**

To evaluate the implementation of high‐throughput NIPT for fetal *RHD* genotype in maternity units in England by addressing research recommendations from the NICE. These were to reduce uncertainty around the resource use and cost of staff training, management of samples and results and record‐keeping, as well as resultant changes to antenatal or post‐partum care and performance of NIPT.

**Methods:**

A cross‐sectional survey was developed and sent to clinicians at 39 English NHS Trusts in May 2018. Qualitative interviews with seven individuals were conducted to explore missing or contraindicatory data. Qualitative findings were supplemented with NIPT test results (April 2017 to February 2019) from English hospitals.

**Results:**

Staff reported that training took up to 30 minutes. There were no extra costs associated with sample management or additional appointments. Extra time required for record‐keeping and management of test results was balanced later in the patient pathway. The antenatal pathway was not changed in the Trusts surveyed. The survey revealed that four post‐partum scenarios were being used within English NHS Trusts. The frequency of inconclusive NIPT results was 4.3%.

**Conclusion:**

NIPT for fetal *RHD* genotype can be implemented without consuming substantial extra resources through incorporation into an existing patient pathway.

## 1 INTRODUCTION

Babies of a Rhesus D (RhD)‐negative mother can inherit the RhD‐positive blood type from their father. This type of pregnancy is at high risk of sensitisation where anti‐D antibodies develop against RhD antigens. Sensitisation can lead to haemolytic disease of the fetus/newborn or even stillbirth.^1^, ^2^

### 1.1 *Current antenatal pathway for RhD‐negative women*

In August 2008, the National Institute for Health and Care Excellence (NICE) recommended routine antenatal anti‐D Prophylaxis (RAADP) for RhD‐negative women who are not sensitised to the RhD antigen.^2^

For uncomplicated pregnancies, RAADP is given to all RhD‐negative women at or around 28 weeks' gestation (and at 34 weeks if given in two doses) as part of a standard schedule of appointments and following a sensitising event (eg, abdominal trauma, invasive intrauterine procedure etc). The blood type of a baby is determined with cord blood testing (CBT), and potential feto‐maternal haemorrhage is estimated with the Kleihauer test. If a child is confirmed as RhD‐positive, the RhD‐negative mother will receive another dose of RAADP to prevent complications in future pregnancies.

Within the United Kingdom, approximately 40% of RhD‐negative women carry RhD‐negative fetuses; in such cases, there is no risk of haemolytic disease of the foetus/newborn. These women receive RAAPD (an injection of a blood‐related product, which is associated with risk of blood‐borne infections) unnecessarily.^2^

### 1.2 *Introduction of non‐invasive prenatal testing for fetal RHD genotype*

**FIGURE 1:**
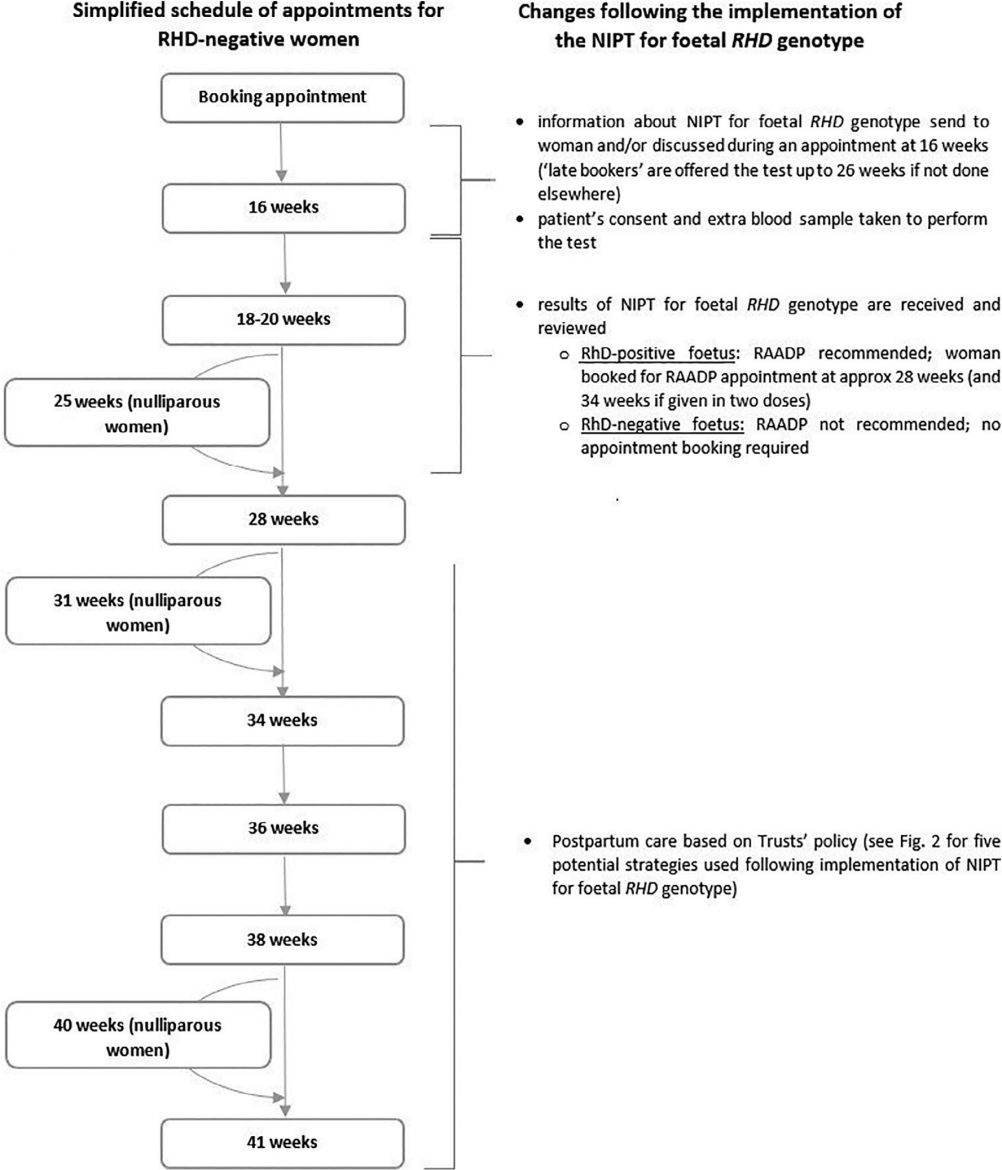
Simplified schedule of appointments based on the current antenatal pathway in the United Kingdom for women known to be RhD‐negative (according to results of blood test at booking appointment) with highlighted changes to the pathway following the implementation of non‐invasive prenatal testing (NIPT) for fetal RHD genotype. Please note that each Trust can introduce changes to the schedule of appointments

In 2016, NICE recommended high‐throughput non‐invasive prenatal testing (NIPT) for fetal *RHD* genotype as a clinically effective and cost‐effective option to guide RAADP in the United Kingdom^3^ (Figure 1). NIPT is offered during the early stages of pregnancy in a routine antenatal appointment and enables the assessment of fetal Rh status from a maternal peripheral blood sample. Based on the published literature evaluating the test performance at different time points, the false negative rate is much higher before 11 weeks and becomes stable after this time^4^, ^5^; thus, the test is more accurate after the first trimester of pregnancy. The results inform whether RAADP is required during pregnancy. The new testing strategy is used as an addition to current standard care, and it has the potential to remove the need for CBT in the future.

These recommendations from NICE are likely to impact staff by requiring additional training of midwives and laboratory staff in order to safely perform the test and manage the sample, extra blood sampling and an increase in administrative tasks (taking consent and checking results). However, the new service is expected to affect the total number of RAADP doses administered and improve patients experience through, for example, reduction in the anxiety associated with the injections. In NICE's guidance, 12 studies were identified in a review of implementation of NIPT for fetal RHD.^3^ Most concluded that implementation of this new technology was feasible, but issues included anti‐D prophylaxis adherence, the importance of short transport times for samples and effective management of sample transport, and a need for greater knowledge of NIPT among physicians and midwives.

NICE identified uncertainties in its guidance and recommended data collection and analysis by Trusts implementing the service associated with costs and resource use of providing the NIPT service in practice. The recommendations were focused on staff training, informing patients, sample management, record‐keeping, management of results, changes to antenatal or postnatal pathways, test failures and adherence to new test and RAADP.^3^ The aim of this study was to evaluate the implementation of NIPT for a fetal *RHD* genotype by addressing NICE's research recommendations, specifically the following objectives: (a) to assess the length and arrangements for staff training; (b) to assess costs and management of samples; (c) to identify administrative tasks that require extra time; (d) to evaluate management of mislabelled samples, inconclusive results and test results; (e) to evaluate changes to schedule and timing of antenatal appointments; (f) to establish what are the current post‐partum strategies; and (g) to report on performance of the test.

## 2 MATERIALS AND METHODS

This was a cross‐sectional observational survey of a maternity unit and associated staff in the United Kingdom, supplemented with semi‐structured interviews with clinical experts and routinely collected laboratory results. These three elements (phases) of the study have been described in separate sections below. Discussions with the Cardiff & Vale University Health Board Research & Development department confirmed that these activities were not researches, and as such, ethical approval was not sought.

### 2.1 *Phase 1: Survey*

A questionnaire was developed based on addressing the research recommendations included in NICE's Diagnostics Guidance on NIPT for fetal RHD genotype (DG25).^3^ The areas of uncertainty were formulated into questions that could be answered by clinicians. The draft questionnaire was sent to four experts, and their comments were incorporated into the next version of the document and sent for a second check before the development of the final version (see Table S1). The questionnaire included 18 open and 3 closed questions, accompanied by diagrams for post‐partum scenarios, and was divided into sections: (a) consent and general information, (b) implementation of NIPT for *RHD*, (c) antenatal care, (d) NIPT for *RHD* genotype and RAADP adherence and (e) post‐partum care.

The target sample was clinicians (midwives, transfusion practitioners or haematologists) involved in the implementation of NIPT for *RHD* genotype in 39 English NHS Trusts. The list of Trusts was provided by the International Blood Group Reference Laboratory (IBGRL), the leading laboratory responsible for processing NIPT for fetal *RHD* genotype samples in England. Clinicians' details were identified by an online search and by personal communication with IBGRL. To evaluate the implementation locally, this study included only NHS Trusts from England.

The survey was distributed by email between May and June 2018. The responses were provided through an online survey, e‐mail or a phone call. Reminders were sent after 2 and 4 weeks. Responders were asked to indicate consent to be contacted in the next stage of the study (expert interviews). The analysis was performed in Microsoft Excel 2013 by one researcher (E.R.)—qualitative and quantitative data were categorised by question, checked for any errors and summarised in a narrative.

### 2.2 *Phase 2: Expert interviews*

The results from the survey were used to identify missing or contradictory information requiring more in‐depth discussion. Phase 2 of the study used semi‐structured interviews with clinical experts. A guide sheet with the same structure as the survey was developed and sent to clinical experts prior to the interview to allow them to familiarise themselves with topics and consider their responses (see Table S2). To ensure a good geographical spread, a range of interviewees from both the north and the southeast of England were invited. Each interview lasted approximately 60 minutes, and it was recorded following participants' verbal consent. Transcripts were written verbatim by E.R. The transcripts were sent, verified and corrected by interviewees within 2 weeks of the interview. Analysis of the qualitative data was performed in Microsoft Word 2013 using the thematic techniques^6^—answers were categorised by relating to the same question asked, reviewed, each category given a title and summarised in the narrative.

### 2.3 *Additional data collection*

IBGRL provided results of all NIPT for fetal *RHD* genotype samples (positive, negative, inconclusive and not tested) from laboratories across England that were tested between April 2017 and February 2019. No patient‐identifiable data were shared.

During preparation of this manuscript, the authors used the consolidated criteria for reporting qualitative research^7^ and critical appraisal skills programme checklists^8^ for reporting of qualitative studies.

## 3 RESULTS

Overall, staff from 25 of 39 (64%) NHS Trusts who were sent surveys provided a response. Respondents completed an average of 13 of 15 questions in the survey, excluding the first section which contained administrative questions only. The response rate varied between questions; the number of responses received is stated in each subsection below. Questions with more than a 50% response rate, including data than can be categorised into emerging theme, are reported in this article. Uptake and adherence to either the NIPT or RAADP were estimates and/or clinicians' opinion, not actual data, and they are not included in the results.

At the time of survey completion, 21 of 25 survey responders (84%) indicated that their Trust had already implemented the NIPT for fetal RHD genotype testing. The remaining sites were preparing for implementation. The qualitative interviews were carried out with seven clinicians from three high‐volume (>5000 deliveries per year) and three lower‐volume (<5000 deliveries per year) NHS centres.

Results from the survey and interviews have been presented together under subsections that correspond to objectives of the study.

### 3.1 *Aim 1: Length and arrangements for staff training*

**FIGURE 2:**
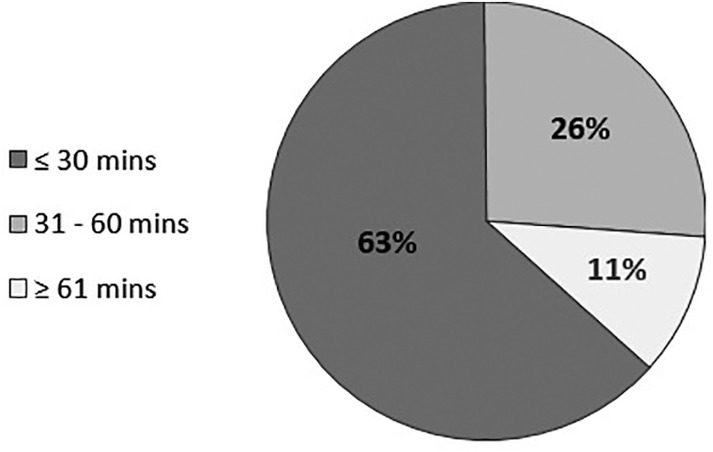
The estimated training time per healthcare professional based on the survey responses provided by Trusts (n = 19 Trusts)

Training in NIPT service provision was estimated by 12 of 19 survey respondents (63%) to take 30 minutes or less (Figure 2). According to survey responses, nurses and midwives, followed by laboratory staff and doctors, were most likely to receive the training. For existing employees, internal training utilised already‐scheduled sessions, such as mandatory training days or other meetings, to briefly review procedures used and familiarise themselves with the expected addition in practice. Updated policy flowcharts, Standard Operating Procedures and guidelines were used as the main source of information. For new employees, information was included during specialised induction sessions in the area.

Interviewees highlighted that a lack of compatibility between the electronic reporting system used by IBGRL and local laboratory IT system resulted in some staff requiring an extra training session to provide information on how to access the NIPT for fetal *RHD* genotype reports and record patient results.

### 3.2 *Aim 2: Costs and management of samples*

Of 16 survey responders, 10 (62.5%) reported that there were no extra costs or cost savings associated with the transport and management of samples. Currently, only one centre (IBGRL) receives and analyses the samples. None of the Trusts required extra arrangements with IBGRL in order to collect and transport samples as all specimens are sent immediately after collection via an existing route. Interviews revealed that special delivery arrangements are required before long weekends or bank holidays in order to maintain sample validity.

### 3.3 *Aim 3 and 4: Record‐keeping and management of results*

Of 17 survey responders, 11 (65%) stated that administrative tasks before and after the sample collection required extra time. Importantly, interviews revealed that there is no uniform IT system between the IBGRL and local hospital. The tasks included (a) recording patients' decision and consent for the test, (b) manual transcribing results between IT systems and into patients' notes, (c) informing patients about results and further steps, (d) follow up of results and unprocessed samples and (e) audit.

Lack of compatibility between the IBGRL and local IT systems was estimated by interviewees to require an extra 5 minutes per patient for transfer of results.

Interviews and survey results showed that samples mislabelled due to human error were rejected in the laboratory, and women were informed about the possibility of having another test sample taken. In most cases, inconclusive test results are treated as RhD‐positive, and women will receive RAADP later in pregnancy.

The results of NIPT for fetal *RHD* genotype are recorded in the patient's electronic record with automatic comments for midwives as a sticker in the baby's notes or as a paper copy in their notes, sometimes including the patient pathway.

### 3.4 *Aim 5: Changes to antenatal care*

All survey responders reported that the patient pathway does not require any extra appointments. Women are informed about the test before or during their routine 16‐week appointment in the form of written materials or discussion with a healthcare professional.

Of 17, 10 (59%) survey responders reported that appointments do not require extra time. Sample collection is incorporated into an existing appointment at the 16th week of pregnancy. Of 17 responders, 7 (41%) stated that appointment can be slightly longer due to administrative tasks estimated to take between 3 and 15 minutes.

Women eligible for NIPT for *RHD* genotype but presenting to hospital after the time when the test is offered (“late bookers”) can access the service only if they did not receive it elsewhere. In most Trusts, the NIPT for *RHD* genotype is offered up to the 26th week of pregnancy, just before RAADP appointment.

Of 18, 13 (71%) survey responders stated that some appointments are shorter or not required. Women with a predicted RhD‐negative baby do not need RAADP; thus, fewer RAADP appointments at 28 weeks' gestation, and following any sensitising event, are needed.

### 3.5 *Aim 6: Changes to post‐partum care*

**FIGURE 3:**
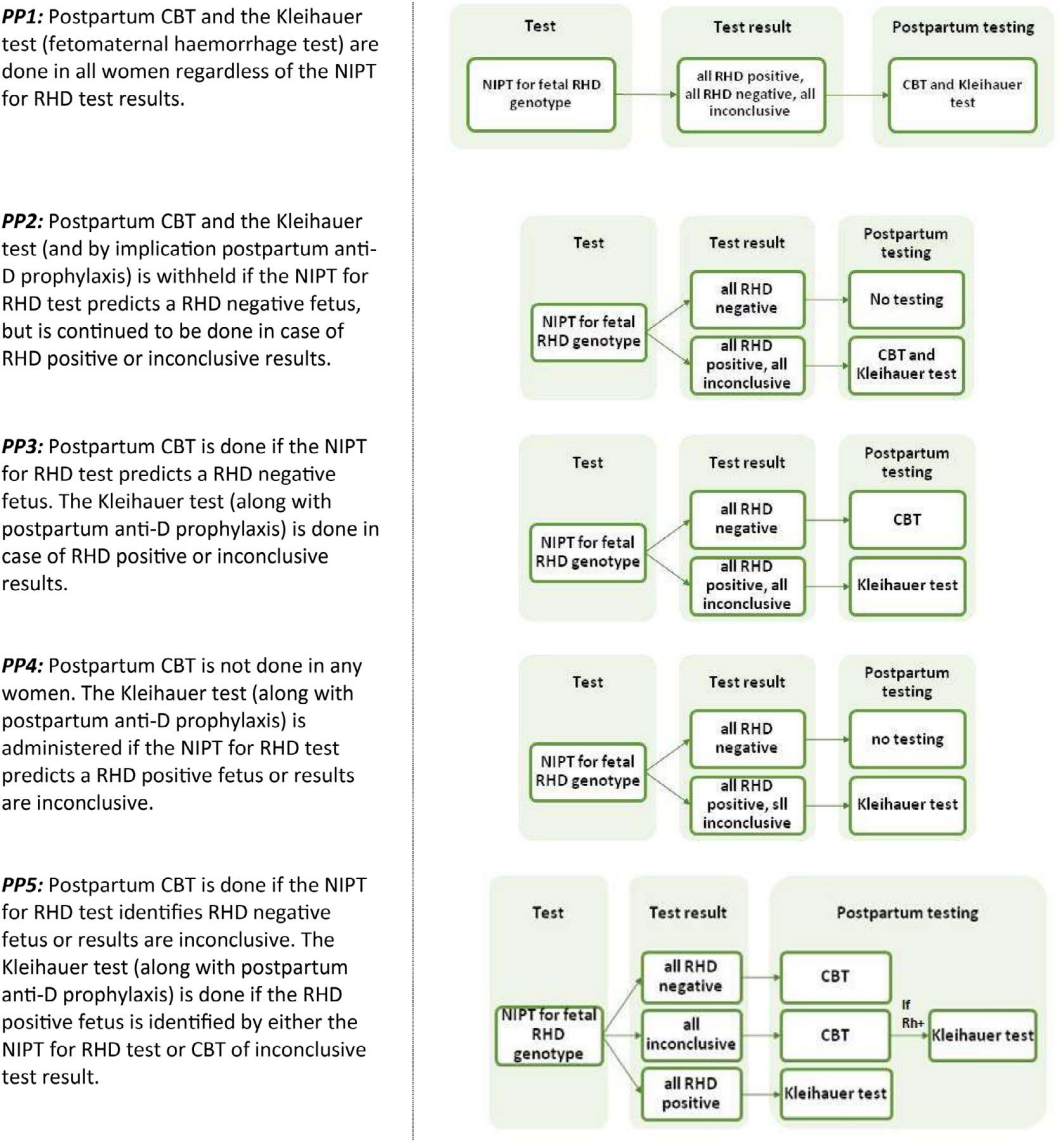
Five potential post‐partum strategies used in Trusts following implementation of non‐invasive prenatal testing (NIPT) for fetal RHD genotype

Five different potential post‐partum scenarios (PP) were investigated (PP1‐PP5, see Figure 3) based on 19 survey responses received. Of 19, 6 (32%) reported using PP5, 5 (26%) PP1 and 4 (21%) PP3. Scenario PP5*, a modified PP5, is used in 4 of 19 Trusts (21%). In this scenario, CBT is performed for all babies despite the results of NIPT for fetal *RHD* genotype. The Kleihauer test is performed for predicted or confirmed RhD‐positive babies only.

The simplified schedule of appointments for women known to be RHD‐negative with incorporated findings in this study can be seen in Figure 1.

### 3.6 *Aim 7: Performance of the NIPT for fetal RHD genotype*

**FIGURE 4:**
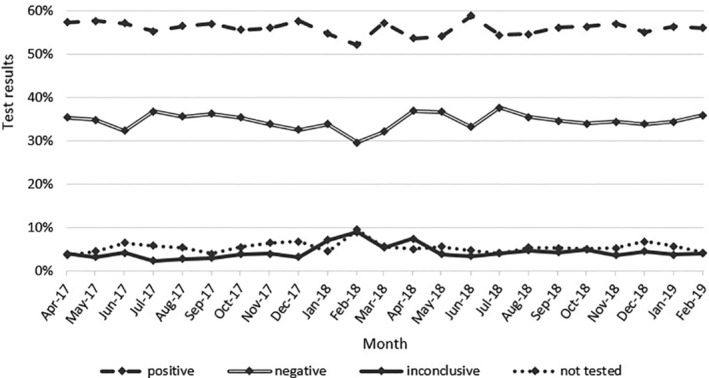
The results of non‐invasive prenatal testing (NIPT) for fetal RHD genotype tests (%) from all hospitals in England that implemented the new testing in each month between April 2017 and February 2019. The data were provided by International Blood Group Reference Laboratory (IBGRL)

On average, between April 2017 and February 2019, there were 55.9% positive, 34.5% negative and 4.3% inconclusive results of NIPT for fetal *RHD* genotype. Approximately 5.4% of samples were not tested due to sample issues such as mislabelling of test tubes. The test results per each month are presented in Figure 4.

## 4 DISCUSSION

This article reports the outcomes of a study focusing on the implementation of NIPT for fetal *RHD* genotype in a representative sample of English NHS Trusts. Following the publication by Soothill et al,^9^ evaluating the implementation of the NIPT for fetal *RHD* genotype in three maternity services in England, this study provides more details regarding staff training, sample management, record‐keeping, management of results and changes to antenatal or postnatal pathways. The information is relevant to all maternity units within the United Kingdom and can be translated into other healthcare systems.

NICE investigated the cost‐effectiveness of post‐partum scenarios in a full economic evaluation, including de novo economic modelling, of the NIPT for fetal *RHD* genotype. Saramago et al^10^ concluded that targeted use of anti‐D prophylaxis using NIPT is cost saving compared with current practice of giving anti‐D to all RhD‐negative women. All four scenarios (PP1‐PP4) included in NICE's guidance were cost saving but less clinically effective (resulting in an increase in sensitisations) when compared to standard practice (RAADP administration to all RhD‐negative women). The extent of the estimated cost savings was large enough to outweigh the small quality‐adjusted life year (QALY) reduction (cost savings ranged from £493 000 to £762 000 per 100 000 pregnancies depending on which post‐partum strategy was used). The results of the model were heavily influenced by the cost of both the NIPT test and RAADP. Cost savings could only be achieved when the cost of NIPT was £24 or less. A fifth post‐partum strategy, PP5, which distinguishes between inconclusive results and positive results, was found to be more cost saving than all other strategies.^10^ ^.^The economic evaluation was unable to include all of the benefits of NIPT for fetal *RHD* genotype, such as unnecessary administration of RAADP to RhD‐negative women with RhD‐negative fetuses. This study showed that PP5 is most often used within Trusts in United Kingdom; however, an additional modified scenario, PP5, reported as being in use in some Trusts, needs to be investigated for its cost implications and effectiveness.

Saramago and colleagues'^10^ economic evaluation and the NICE committee found that there was uncertainty regarding the cost of introducing NIPT into routine practice in the NHS. Additional costs associated with blood draws, transport of samples and antenatal visits to deliver test results and counselling could result in NIPT becoming cost‐incurring and therefore no longer the preferred strategy.

Results of this study show that implementation of the NIPT for fetal *RHD* genotype is not associated with an increase in resource use in most of the Trusts. Most Trusts reported no additional costs or resource use associated with transport and management of samples and that no additional antenatal visits were required. However, extra staff time was required to record and manage results. The training and education for staff was reported to take around 30 minutes. The new service has the potential to greatly reduce unnecessary RAADP administration and consumables and improve the quality of care. The extra time needed before and after sample collection is balanced with fewer clinic visits needed at the time of RAADP administration or following a potential sensitising event.

The rate of inconclusive results reported in this article is lower when compared to samples tested in the same laboratory (IBGRL) in Bristol in the United Kingdom, at similar gestational age (beginning of the second trimester) (7.0%; data collected between 2009 and 2012^4^ and 12.2%; April‐September 2013^9^). Despite variations that can be attributed to potential imbalances in characteristics of study populations, the differences in the rates of inconclusive results are considerable.

Interviewees highlighted that assistance of experienced healthcare professionals at early stages of implementation and engagement of employees were crucial factors contributing to successful implementation. Moreover, a routine audit of the clinical practice will enable the capture of any arising and ongoing issues, which can be resolved quickly.

It is important to highlight the risks associated with a lack of uniform reporting system between the laboratory performing the test and local IT. The need to transfer the results between two different platforms carries the risk of transcription errors, which can have a significant impact on patients' health (due to, eg, missed RAADP dose) and contribute to an increase in resource use. The authors encourage discussion within Trusts to determine the best arrangements to transfer the results with the least risk of transcription errors. Moreover, the differences in the patient pathways (eg, schedule of visits) or RAADP management (eg, dosage and time of administration) between hospitals within the same Trust can contribute to delays in incorporating the new service in practice. Each Trust needs to routinely evaluate its current working system in order to incorporate changes without consuming additional resources.

This study has some limitations. The survey and interview methodology was a basic cross‐sectional design, and the sample size was relatively small. Authors relied on clinicians' opinion and level of details provided; interpretation and the data collection was performed by only one researcher. The number of Trusts implementing the NIPT for fetal *RHD* genotype in the United Kingdom is currently increasing; thus, real‐time mapping of the patient pathway and monitoring of the service are suggested as potential research recommendations.

The study was performed shortly after the publication of new recommendations included in DG25. Thus, Trusts were at different stages of implementation, and the new service was not yet embedded in practice, which affected the quality and inaccuracies of responses. The study can be repeated when NIPT for fetal *RHD* genotype is widely used in clinical practice to compare the results and highlight its impact.

The uptake of NIPT for fetal *RHD* genotype, RAADP adherence and false‐negative/‐positive results are not routinely monitored; thus, this article does not report any analysis as responses received were not representative of the population. It is recommended that this type of data is collected in order to fully assess the impact of the new testing strategy. Monitoring of discrepancies between NIPT for fetal *RHD* genotype and CBT can inform future decisions regarding removing CBT from the current postnatal pathway. In addition, the results show that at least 34.5% of women will not require RAADP; thus, the cost implications of implementation of NIPT for fetal *RHD* genotype should be documented.

NICE guidance recommending NIPT for fetal *RHD* genotype in the NHS identified uncertainties regarding the costs associated with implementing the service. This article presents a “real‐world” qualitative study of the implementation of the NIPT service in NHS maternity units in England. Trusts report that implementation of this service does not consume additional resources through management of samples or additional antenatal visits and that the new service fits well within the previous patient pathway. Extra resources may be required to manage and record results and for internal staff training of nurses, midwives, laboratory staff and doctors. NIPT for fetal *RHD* genotype is an important improvement in patient care, which enables targeted prophylactic administration of RAADP only to women who need it. The results of this study reduce much of the uncertainty associated with NICE's recommendation for the routine use of NIPT for fetal *RHD* genotype in the United Kingdom, giving support to the findings that the technology is a cost‐effective option to guide the administration of RAADP.

## CONFLICT OF INTEREST

Cedar, a department within NHS, receives funding from the National Institute for Health and Care Excellence (NICE). NICE not involved in the data collection and analysis. No other conflicts of interest exist.

## Supporting information


**Table S1** The questions included in the survey to clinicians. Please note that question 21 relates to all post‐partum scenarios from Figure 3

**Table S2** The topic guide used during the interviews with clinical experts
