# Supplementary figures and images for: Implementation of high‐throughput non‐invasive prenatal testing for fetal RHD genotype testing in England: Results of a cross‐sectional survey of maternity units and expert interviews
Source: Transfus Med. 2020 Jun 11;30(4):287–94. doi: 10.1111/tme.12702 (PMC7496714; doi:10.1111/tme.12702)

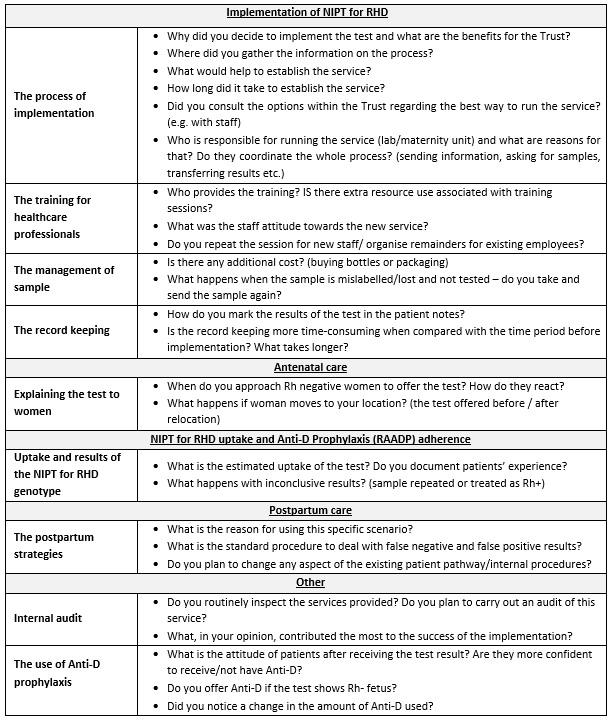

Supplement: Supplementary file 1 — Table S1 The questions included in the survey to clinicians. Please note that question 21 relates to all post‐partum scenarios from Figure 3 [file TME-30-287-s001.jpg]

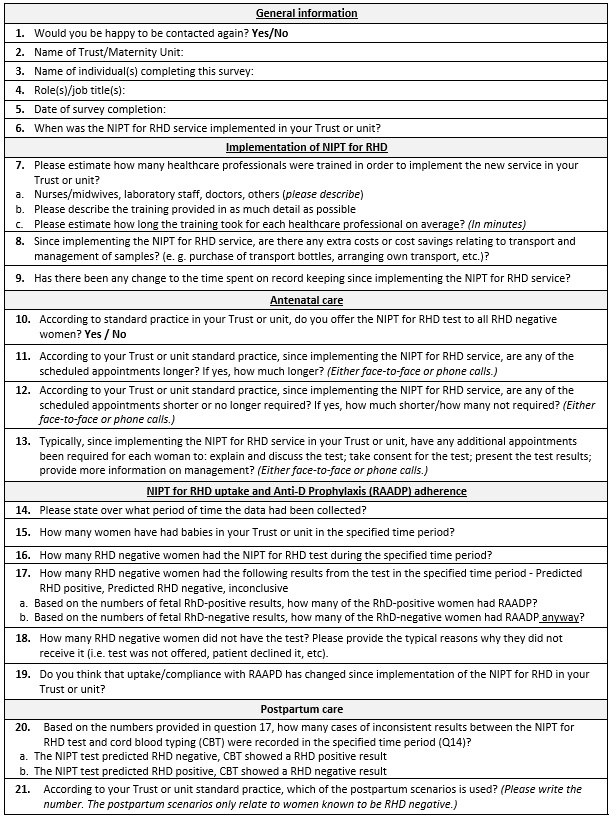

Supplement: Supplementary file 2 — Table S2 The topic guide used during the interviews with clinical experts [file TME-30-287-s002.jpg]
